# Rapid Drug Susceptibility Testing of Drug-Resistant Mycobacterium tuberculosis Isolates Directly from Clinical Samples by Use of Amplicon Sequencing: a Proof-of-Concept Study

**DOI:** 10.1128/JCM.00535-16

**Published:** 2016-07-25

**Authors:** Rebecca E. Colman, Julia Anderson, Darrin Lemmer, Erik Lehmkuhl, Sophia B. Georghiou, Hannah Heaton, Kristin Wiggins, John D. Gillece, James M. Schupp, Donald G. Catanzaro, Valeriu Crudu, Ted Cohen, Timothy C. Rodwell, David M. Engelthaler

**Affiliations:** a Pathogen Genomics, Translational Genomics Research Institute, Flagstaff, Arizona, USA; b Division of Pulmonary, Critical Care, and Sleep Medicine, University of San Diego, San Diego, California, USA; c College of Education and Health Professions, University of Arkansas, Fayetteville, Arkansas, USA; d Phthisiopneumology Institute (PPI), Chisinau, Republic of Moldova; e Yale School of Public Health, New Haven, Connecticut, USA

## Abstract

Increasingly complex drug-resistant tuberculosis (DR-TB) is a major global health concern and one of the primary reasons why TB is now the leading infectious cause of death worldwide. Rapid characterization of a DR-TB patient's complete drug resistance profile would facilitate individualized treatment in place of empirical treatment, improve treatment outcomes, prevent amplification of resistance, and reduce the transmission of DR-TB. The use of targeted next-generation sequencing (NGS) to obtain drug resistance profiles directly from patient sputum samples has the potential to enable comprehensive evidence-based treatment plans to be implemented quickly, rather than in weeks to months, which is currently needed for phenotypic drug susceptibility testing (DST) results. In this pilot study, we evaluated the performance of amplicon sequencing of Mycobacterium tuberculosis DNA from patient sputum samples using a tabletop NGS technology and automated data analysis to provide a rapid DST solution (the Next Gen-RDST assay). One hundred sixty-six out of 176 (94.3%) sputum samples from the Republic of Moldova yielded complete Next Gen-RDST assay profiles for 7 drugs of interest. We found a high level of concordance of our Next Gen-RDST assay results with phenotypic DST (97.0%) and pyrosequencing (97.8%) results from the same clinical samples. Our Next Gen-RDST assay was also able to estimate the proportion of resistant-to-wild-type alleles down to mixtures of ≤1%, which demonstrates the ability to detect very low levels of resistant variants not detected by pyrosequencing and possibly below the threshold for phenotypic growth methods. The assay as described here could be used as a clinical or surveillance tool.

## INTRODUCTION

Globally, tuberculosis (TB) infects an estimated 9.6 million people and is the cause of 1.5 million deaths per year (1). In some regions, such as Belarus, Kazakhstan, the Republic of Moldova, and Uzbekistan, it is estimated that 23 to 34% of new cases and 58 to 69% of retreatment cases are multidrug-resistant TB (MDR-TB), defined by resistance to at least isoniazid and rifampin (1, 2). This high prevalence of drug-resistant TB (DR-TB) illustrates the need for comprehensive drug susceptibility profiling prior to treatment initiation to alleviate and potentially avoid high rates of treatment failure and mortality from undetected DR-TB. Current diagnostic tools for broad drug susceptibility testing (DST) of Mycobacterium tuberculosis isolates are culture based, slow (take weeks to months), not standardized, and require complex laboratory infrastructure (3–5). These complexities lead to systematic errors in the resistance profiles obtained.

Rapid and early detection of drug resistance is essential to guide appropriate treatment, with individualized drug resistance profiles as an ideal. Molecular methods offer an opportunity for rapid drug resistance identification, as demonstrated by the introduction of the Xpert MTB/RIF assay (Cepheid, Sunnyvale, CA), which improved the identification of rifampin resistance and MDR-TB (1). Currently, there is no WHO-endorsed commercially available molecular diagnostic for second-line antituberculosis drug susceptibility testing (1). Non-sequencing-based molecular tests, like line probe assays (6–9) and real-time PCR methods (10, 11), struggle to capture complex resistance profiles and have a limited adaptability to accommodate new genetic mechanisms of resistance as they are discovered. In addition, as the knowledge of resistance mutations has increased, the validation of highly multiplex molecular assays (those addressing extensively drug-resistant TB [XDR-TB], which is MDR-TB with additional resistance to fluoroquinolones and amikacin, kanamycin, or capreomycin) will be increasingly complex. In contrast, next-generation sequencing (NGS) is much simpler to validate for the detection of multiple resistance mutations in M. tuberculosis, as its performance does not depend the number of mutations present.

Significant and continued reductions in NGS costs and operational complexity over the past few years have made routine use of NGS for targeted and whole-genome sequencing (WGS) commonplace in high-income countries and broadly expanded its utility as a viable public health and clinical tool. WGS has previously been employed clinically to examine TB disease spread and identify outbreak situations (12–18). However, WGS currently relies on DNA from TB patient cultures, keeping the approach one step away from where it is needed, i.e., as an unbiased method for rapidly detecting and characterizing clinically relevant DR mutations directly from clinical samples (19). A WGS approach is not currently cost-effective or efficient for TB control, and it is both labor and computationally intensive when done without first culturing an isolate. Utilizing a targeted NGS (amplicon NGS) approach, we can combine the benefits of sequencing large targets (hundreds of nucleotides) with a large depth of coverage (e.g., 10,000× coverage, which is critical for accurate detection of heteroresistance or mixed populations) using DNA obtained directly from patient samples, without the need for isolation of bacteria or removal of human DNA (20). Heteroresistance or mixed populations can complicate the interpretation of molecular resistance test results and may result in indeterminate or false-negative (i.e., drug-susceptible) results when the resistant population makes up less than 10 to 20% of the total population (21–24). While the clinical relevance of resistant subpopulations is still being determined, it appears that early detection of resistant infections could be important to the early implementation of appropriate drug regimens (25, 26). A targeted sequencing approach can be highly sensitive, generate relevant data (i.e., amplicon sequence data versus single nucleotide polymorphism [SNP] determination), increase accuracy over that with alternate methods (i.e., identify individual resistance mutations and predict MICs), and expand the range of targets tested as additional information is gathered.

Previously, we demonstrated the advancement of amplicon sequencing using overlapping reads to examine subpopulations at extremely low levels of antituberculosis drug-resistant bacteria (20). This pilot study evaluates the performance and clinical utility of rapid amplicon sequencing of DNA from patient sputum samples using a tabletop next-generation sequencing technology and automated data analysis scripts to provide an accessible rapid DST (Next Gen-RDST) solution. Utilizing a well-characterized set of DNA from a previous study, we compared these newly generated Next Gen-RDST results against previous phenotypic DST and pyrosequencing results from the same clinical sputum DNA samples.

## MATERIALS AND METHODS

### Specimens.

All specimens used in this study were obtained from a large multisite clinical observation trial conducted by the Global Consortium for Drug-Resistant TB Diagnostics (GCDD [http://gcdd.ucsd.edu]). Detailed methods and results of that study have been previously published (27, 28). Newly presenting TB patients 5 years of age or older and patients who had treatment failure were recruited for the parent study. Only samples obtained from the Republic of Moldova were used in this study. The use of these specimens was reviewed and approved by institutional review boards at the University of California, San Diego (UCSD; Human Research Protections Program), Republic of Moldova (Ethics Committee of Phthisiopneumology Institute, Public Health Medical Institution), and Yale University, New Haven, CT. In total, 254 remnant DNA samples, extracted directly from patient sputum samples recruited in Moldova, were screened for inclusion in this study; eight of these samples did not have enough sample volume and were excluded from our study.

### Reference method phenotypic DST.

Phenotypic drug susceptibility profiles were previously established for all samples using the MGIT 960 platform with the EpiCenter software, as reported previously (27, 28). The previously generated MGIT 960 (BD Diagnostic Systems, NJ, USA) DST results (27, 28) served as the phenotypic reference standard in this study. All specimens were tested against isoniazid (INH), rifampin (RIF), moxifloxacin (MOX), ofloxacin (OFX), amikacin (AMK), kanamycin (KAN), and capreomycin (CAP), according to standard manufacturer protocols and using WHO-recommended critical concentrations, as previously described (4, 28).

### DNA extraction.

Sample DNA was previously extracted directly from sputum samples from 246 TB patients, as described previously (27, 28). All M. tuberculosis DNA was extracted directly from pooled sputum samples that were decontaminated and concentrated by the *N*-acetyl-l-cysteine (NALC)-NaOH method (29). The pooled sputum samples were a combination of a spot sample collected on the day of enrollment and a sample produced early the following morning.

### Control DNA.

DNA from a confirmed pansusceptible M. tuberculosis strain was used as a resistance sequencing error control throughout the Next Gen-RDST assay. This control DNA was extracted from a culture of a Moldovan strain housed in GCDD's strain bank at the University of California, San Diego (30), identification sample 2-0112. Phenotypic DST and multiple molecular diagnostic tests have confirmed the isolate to be wild type and pansusceptible. Sample 2-0112 was cultured from single-colony isolation and extracted using the Qiagen Genomic-tip with genomic DNA buffer set. The manufacturer's sample preparation for bacteria was used, with a modification to the lysis step as previously described (20). Following lysis, the Genomic-tip protocol in the Qiagen Genomic DNA Handbook was used.

### Pyrosequencing assay.

Previous pyrosequencing data were used to validate our targeted NGS results. All pyrosequencing reactions were conducted previously (27, 28), using the PyroMark Q96 ID system (Qiagen, Valencia, CA), and were performed according to the manufacturer's instructions (31). The *eis* promoter target was added to the platform following the original study completion, and all samples were subsequently sequenced at this target to further characterize KAN resistance.

### Real-time PCR screen for TB DNA.

As the volumes of remnant DNA available for this study were limited, an initial quantitative PCR (qPCR) screen, targeting the *rpoB* gene, was conducted with a 1:7 sample dilution in triplicate to confirm the presence of M. tuberculosis target DNA before the samples were subjected to targeted NGS. The PCR parameters are as follows: uracil-*N*-glycosylate (UNG) activation at 50°C for 2 min, initial denaturation at 95°C for 2 min, 40 cycles of denaturation at 95°C for 15 s, annealing/extension at 60°C for 1 min, and last, a dissociation curve. A single 10-μl qPCR mixture contains 2 μl of DNA, 5 μl of Platinum SYBR green qPCR SuperMix–uracil-DNA glycosylase (UDG) (catalog no. 11733-038; Invitrogen), 1 μl of forward and reverse primer (0.5 μM final concentration), and 1 μl molecular-grade H_2_O. The forward and reverse primers were the *rpoB* gene-specific primers (20).

### Next Gen-RDST assay.

Samples that amplified via qPCR and had a product melting temperature (*T_m_*) of ∼91°C were run through the Next Gen-RDST assay workflow, as described previously (20), with the following modifications. After the gene-specific multiplex PCR, the reaction mixture was cleaned twice, first using a 1× and then a 0.8× Agencourt AMPure XP bead (Beckman Coulter, Brea, CA) cleanup, and the amplicons were eluted in 25 μl of a 10 mM Tris-HCl–0.05% Tween 20 solution. This modification removes short amplification artifacts (i.e., primer dimer) prior to indexing and sequencing. These amplification artifacts occur at significant levels with low M. tuberculosis DNA concentrations, as typically found in patient samples. The gene-specific multiplex PCR contains six gene regions critical for detecting mutations associated with the XDR phenotype: *katG* and the *inhA* promoter to characterize INH resistance, *rpoB* (one amplicon containing both regions examined by pyrosequencing) to characterize RIF resistance, *gyrA* to characterize fluoroquinolone resistance, *rrs* to characterize injectable resistance, and the *eis* promoter to characterize KAN resistance (in addition to the *rrs*) (20). To reduce optical contamination, a dual indexing approach (32) was used by modifying the index extension PCR. The common universal tail primer was replaced with a specific indexed universal tail 2 primer (see Table S1 in the supplemental material). Eighty-eight clinical sample libraries plus a negative and positive (pansusceptible) sequencing control library were pooled for sequencing on one Illumina MiSeq 2 × 300-bp version 3 run. At least 25% of each sequencing run was filled with PhiX control to ensure base diversity and reduce complications with sequencing. In all runs, the pansusceptible isolate was included as a Next Gen-RDST assay error control for the accurate analysis of alternate allele calls at positions of interest.

### Sequencing analysis.

To illustrate the flexibility of analysis on amplicon sequencing, two different bioinformatics tools were used on all the samples for the Next Gen-RDST analysis presented in this study. Either tool could be used individually without the need for other analyses, depending on the results desired. The single-molecule overlapping-read (SMOR) analysis tool was used to examine low-level variation and rare mutations, as previously published (20). Briefly, after removal of adapter sequence with Trimmomatic (33), reads were mapped against amplicon-specific reference sequences (developed from H37Rv accession no. NC_000962; NCBI) using Novoalign (Novocraft) with the default parameters. The SMOR analysis tool (20) automates the process of acquiring counts at a position of interest (i.e., SNP locus). For every read pair collected, a tally is made of the frequency at which each nucleotide appears at that position of interest on both reads. Paired reads that disagree are excluded and considered sequencing errors, due to the fact that the only way for the reads from the same DNA molecule to disagree is sequencing error. This use of overlapping reads allows for low-level subpopulation detection (20). For the purpose of this study, a Next Gen-RDST genotypic call of resistance to a drug was made if a resistant subpopulation was detected at ≥10%, while lower proportions were recorded. The conservative cutoff of 10% for calling of resistance alleles was chosen to enable the comparison of genotypic calls between pyrosequencing and Next Gen-RDST (20, 34).

The second bioinformatics tool used is a newly developed TB Amplicon Sequencing Analysis Pipeline (TB-ASAP) that produces a clinically relevant DST report using the paired-end raw reads from the sequencer and a JavaScript Object Notation (JSON) file describing the assays used in this study. The reads are first trimmed of any adapter sequences and then for quality using a 5-base-wide sliding window, and are cut when the average quality drops below 20, with Trimmomatic (33). Since TB-ASAP does not currently consider whether both reads in an overlapping read pair agree at any given position, as the SMOR analysis does, the quality trimming is important to help reduce spurious calls due to sequencing error. The trimmed reads are then aligned to the target amplicon sequence references using Novoalign (Novocraft), and the resultant BAM files are analyzed alongside the assay descriptions in the JSON file to determine the presence and frequency of any single nucleotide polymorphism (SNP) and the frequency of codon sequences at known antibiotic resistance-conferring positions. User-defined thresholds for depth of coverage and percent subpopulation detection were set to 20× coverage and mixtures of ≥10% for this analysis. TB-ASAP outputs an XML file containing the detailed analysis of each of amplicon target against each of the samples, which can then be converted into several levels of output using extensible stylesheet language transformations (XSLT). The final outputs include a top-level clinical report (Fig. 2) showing the mutations present, subpopulation detection, and drug significance for specific SNP locations, and a mid-level research report, including more details, such as the number of reads that aligned and the base and codon distributions at each of these locations. All pertinent files for TB-ASAP are publically available (https://github.com/TGenNorth/TB-ASAP).

### Nucleotide sequence accession numbers.

All sequencing read files were deposited in the NIH Sequence Read Archive under BioProject no. PRJNA322712.

## RESULTS

### qPCR screening.

**FIG 1:**
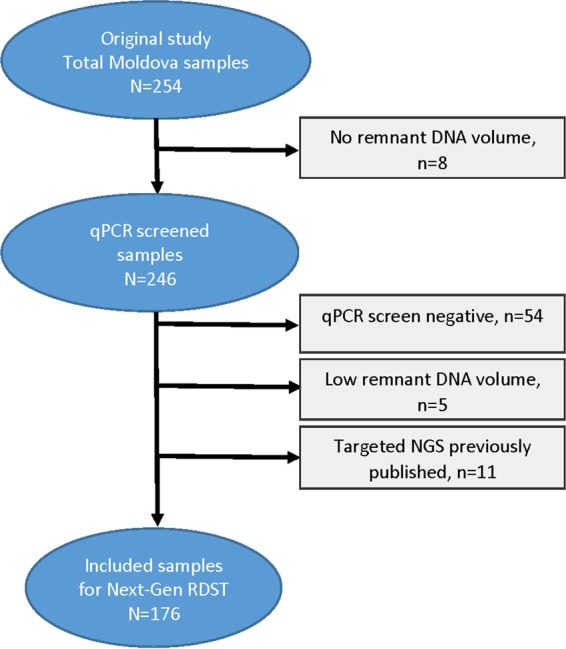
Flowchart of sample inclusion/exclusion for Next Gen-RDST analysis.

M. tuberculosis DNA presence was confirmed in 192/246 (78.0%) samples with amplification in at least one of three replicates of *rpoB*-specific qPCR, with a product *T_m_* of ∼91°C. Fifty-four samples (22.0%) failed across all three replicates of *rpoB*-specific qPCR and thus were not included in the Next Gen-RDST analysis, due to DNA volume constraints dictating that multiple attempts for Next Gen-RDST were not possible. Five of the qPCR-positive samples had extremely low original DNA volumes, and 11 samples had been previously examined with targeted NGS (20); thus, these 16 samples were not included in the Next Gen-RDST assay analysis reported in this study, resulting in a total of 176 samples being included in the Next Gen-RDST analysis (Fig. 1 and Table 1).

**TABLE 1:** Moldova sample breakdown

Smear result	Total sample	No. (%) with screen result:		No. (% of screen positive) with complete data by:	
		Negative^*a*^	Positive	Next Gen-RDST	PSQ^*b*^
Negative	83	50 (60.2)	33 (39.8)	27 (81.8)	8 (24.2)
Positive	171	28 (16.4)	143 (83.6)	139 (97.2)	84 (58.74)
Rare (scanty±)	26	5 (19.2)	21 (80.8)	18 (85.7)	15 (71.43)
Few (1+)	44	7 (15.9)	37 (84.1)	36 (97.3)	22 (59.5)
Many (2+)	63	9 (14.3)	54 (85.7)	54 (100)	26 (48.15)
TNTC (3+)^*c*^	38	7 (18.4)	31 (81.6)	31 (100)	21 (67.75)
Total	254	78 (30.7)	176 (69.3)	166 (94.32)	92 (52.27)

a Eight samples did not have sufficient volume of remnant DNA for screening.

b PSQ, pyrosequencing.

c TNTC, too numerous to count.

### Phenotypic DST.

MGIT 960 phenotypic results were available (28) for 173/176 (98.3%) of the qPCR-positive Moldovan sputum samples examined in this study (see Data Set S1 in the supplemental material). The three samples that failed phenotypic DST (21-0068, 21-0088, and 21-0091) were smear-negative samples and failed across both genotyping methods.

### Pyrosequencing.

Pyrosequencing generated (28) complete data (data across all nine gene regions examined) for 92/176 (52.3%) samples (Table 1). The pyrosequencing data were incomplete among samples collected from smear-negative patients; only eight samples from smear-negative individuals (24.2%) resulted in complete pyrosequencing data (Table 1).

### Next Gen-RDST performance.

Of the samples submitted to Next Gen-RDST, 173/176 (98.3%) resulted in genotyping calls (i.e., TB reads aligning to gene targets). Three smear-negative samples resulted in no reads aligning to the TB gene targets, and thus, no Next Gen-RDST genotype was recorded. All three samples that failed Next Gen-RDST analysis had high threshold cycle (*C_T_*) values (i.e., low target levels) on the qPCR, with one or more of replicates amplifying. These three samples (21-0068, 21-0088, and 21-0091) had a history of poor characterization in the GCDD study and failed to generate information from any of three DST methods attempted (see Data Set S1 in the supplemental material). Seven additional samples had incomplete Next Gen-RDST assay results, where data were obtained for at least one gene target but one or more targets did not yield any data. Overall, the Next Gen-RDST assay succeeded in producing sequence for all targets in 166/176 (94.3%) of the samples, with at least 20 or more reads aligned to all amplicon targets. The Next Gen-RDST assay performed well on both smear-negative and smear-positive samples (Table 1). In total, 27 mutations were found in the complete data set (Tables 2 and 3), 20 of which have been previously identified as high-confidence SNPs associated with antibiotic resistance (35). All the results reported are based on SMOR analysis; however, the two bioinformatic approaches gave nearly identical results (Table 2).

**TABLE 2:** Comparison of Next Gen-RDST-, PSQ-, and TB-ASAP-identified mutations in clinical samples^*a*^

Allele by gene target	No. of calls		
	Next Gen-RDST	PSQ	TB-ASAP
*katG*			
315GGT	94	87	92
315TGT	1	1	1
No mutation	76	70	75
Indeterminate	5	18	8
*inhA*			
−15T	51	43	51
−17T	1	1	1
No mutation	117	116	117
Indeterminate	7	16	7
*rpoB1*			
516TAC	1	0	1
516GTC	1	1	1
No mutation	170	131	166
Indeterminate	4	44	8
*rpoB2*			
531TTG	71	58	71
526AAC	1	1	1
526TAC	1	1	1
531TGG	5	5	3
526TAC and 531TTG	2	0	2
No mutation	92	61	89
Indeterminate	4	50	9
*gyrA*			
95 ACC	139	106	Not examined
94GGC and 95ACC	1	1	1
90GTG and 95ACC	3	2	3
91CCG and 95ACC	3	2	3
94GCC and 95ACC	4	3	4
88GCC and 95ACC	1	1	1
90GTG and 94GGC and 95ACC	1	0	1
No mutation	20	18	157^*b*^
Indeterminate	4	43	6
*rrs*			
1401G	4	4	3
1484T	1	0	2
No mutation	165	156	155
Indeterminate	6	16	16
*eis*			
−12T	47	37	46
−10A	3	3	3
−14T	2	1	2
−37T	1	1	1
No mutation	115	129	113
Indeterminate	8	5	11

a Examining only previously identified SNPs that confer resistance.

b Contains samples with 95ACC mutations.

**TABLE 3:** Other mutations identified with Next Gen-RDST

Gene	Codon (WT→MUT)^*a*^	Sample no.	Notes
*katG*	293ATC→GTC	22-0115	Has *katG* 315GGT mutation, phenotypic DST resistant
*katG*	305CCG→ACG	21-0059	Has *katG* 315GGT mutation, phenotypic DST resistant
*rpoB*	518AAC→AGC	21-0025	Has *rpoB* 516TAC mutation, phenotypic DST susceptible
*rrs*	1407 T→C	22-0115	Has *eis* −12T mutation, phenotypic DST resistant to CAP and KAN (CAP is discordant with −12T)
*rrs*	1414 C→T	21-0018	Has *eis* −12T mutation, phenotypic DST resistant to AMK, CAP, and KAN (AMK+CAP is discordant with −12T)
*rrs*	1443 C→G	22-0121, 22-0022	No other mutations in *rrs* or *eis*, phenotypic DST susceptible

a WT, wild type; MUT, mutation.

### Next Gen-RDST as predictor of phenotype.

The concordance between Next Gen-RDST and phenotypic DST was high among all the antibiotics examined, with correlations ranging from 94.6 to 98.8%. The Next Gen-RDST assay performed well for the direct detection of INH, RIF, and KAN resistance, with specificities of 100%, 98.9%, and 93.9% and sensitivities of 95.0%, 97.6%, and 96.2%, respectively (Table 4). For fluoroquinolone (FQ) resistance detection (OFX and MOX), the sensitivity and specificity ranges were 85.7 to 86.7% and 99.4 to 100%, respectively (Table 4). For the detection of resistance to AMK and CAP, specificity was high (∼98%), but sensitivities were low, at 42.9% and 44.4%, respectively (Table 4). Very major errors, defined as phenotypically resistant with no mutation found, ranged from 1 to 3%. Major errors, defined as phenotypically susceptible with a high-confidence SNP associated with resistance found, ranged from 0 to 4% (Table 4).

**TABLE 4:** Summary of Next Gen-RDST results from clinical specimens^*a*^

Mutations detected with Next Gen-RDST by antibiotic (*n*)	No. with phenotypic DST result of^*b*^:		Accuracy analysis results (% [95% CI])^*c*^		
	R	S	Correlation	Sensitivity	Specificity
INH (171)					
Mutations detected	95	0	97.1	95.0 (88.2–98.1)	100.0 (93.6–100)
No mutations	5^*d*^	71			
RIF (172)					
Mutations detected	81	1	98.3	97.6 (90.8–99.6)	98.9 (93.0–99.9)
No mutations	2^*e*^	88^*f*^			
AMK (166)					
Mutations detected	4	3^*g*^^,^^*h*^	95.2	44.4 (15.3–77.3)	98.1 (94.1–99.5)
No mutations	5	154			
CAP (166)					
Mutations detected	3	2^*g*^^,^^*h*^	96.4	42.9 (11.8–80.0)	98.7 (95.1–99.8)
No mutations	4	157			
KAN (166)					
Mutations detected	50	7^*h*^	94.6	96.2 (85.7–99.3)	93.9 (87.3–97.3)
No mutations	2	107			
MOX (172)					
Mutations detected	13	0	98.8	86.7 (58.4–97.7)	100.0 (97.0–100)
No mutations	2	157			
OFX (172)					
Mutations detected	12	1	98.3	85.7 (56.2–97.5)	99.4 (96.0–100)
No mutations	2	157			

a Results only include detection of previously identified high-confidence SNPs associated with resistance (i.e., the category of no mutations refers to zero high-confidence SNPs identified).

b R, resistant; S, susceptible.

c CI, confidence interval.

d One sample had a mixture, with 12% resistant at *rrs* 1401G.

e One sample had very low coverage (56×), with 96% of reads having a susceptible allele.

f One sample has an additional mutation in *rpoB*.

g One sample had a low-level mixture, where 2% of reads were of a resistant mutation.

h One sample had a mixture, with 16% resistant at *rrs* 1484T.

### Comparison of Next Gen-RDST and pyrosequencing.

The number of samples available for the Next Gen-RDST assay and pyrosequencing comparison varied by gene target, due to amplification failures in both methods. The summary results for the specimens are shown in Table 2. Overall, the results were in high agreement; 988/1,010 (97.8%) of mutation target comparisons were concordant (Table 5) between the Next Gen-RDST and pyrosequencing methods. When taking into account the phenotypic DST data as well, Next Gen-RDST results that were discordant with pyrosequencing were consistent with the phenotype for 20 of 23 discordant mutations found between the two genotypic methods (Table 5). The majority (*n* = 10) of discordant results between the Next Gen-RDST assay and pyrosequencing were in the *eis* promoter target, with the other targets displaying no more than 2% discordance (0 to 3 samples each).

**TABLE 5:** Discordances between Next Gen-RDST and PSQ^*a*^

Gene	Sample no.	SMOR result(s) (mutation, coverage at position ×, % with mutation)	PSQ result	Phenotypic DST result
*katG*	21-0096	315GGT, 54, 100	No mutation	R
*katG*	22-0116	315GGT, 6,538, 14	No mutation	R
*inhA*	21-0001	−15T, 7,822, 13	No mutation^*b*^	R
*inhA*	21-0004	−15T, 3,404, 12	No mutation^*b*^	R
*rpoB2*	21-0001	526TAC, 4,814, 10; 531TTG, 4,792, 81	531TTG	R
*rpoB2*	21-0004	526TAC, 1,348, 18; 531TTG, 1,338, 66	531TTG	R
*rpoB2*	21-0030	531TTG, 30,016, 100	No mutation	R
*rpoB2*	22-0112	531TTG, 42,296, 100	No mutation	R
*rpoB2*	22-0116	531TTG, 3,888, 22	No mutation	R
*gyrA*	21-0049	90GTG, 27,802, 63; 94GGC, 28,530, 36	No mutation	R
*rrs*	22-0122	No mutation (A), 320, 100	1401 G	S
*rrs*	21-0021	1401G, 696, 16	No mutation	S
*rrs*	21-0004	1401G, 3,532, 10	No mutation^*c*^	S
*eis*	21-0054	−12T, 5,550, 100	No mutation	R
*eis*	21-0055	−12T, 52, 100	No mutation	R
*eis*	21-0059	−12T, 278, 100	No mutation	R
*eis*	21-0082	−14T, 1,158, 100	No mutation	S
*eis*	21-0085	−12T, 976, 100	No mutation	R
*eis*	21-0093	−12T, 1,174, 100	No mutation	R
*eis*	22-0015	−12T, 4,292, 74	No mutation	R
*eis*	22-0072	−12T, 3,220, 100	No mutation	R
*eis*	22-0126	−12T, 4,320, 100	No mutation	R
*eis*	23-0069	−12T, 712, 100	No mutation	R

a Discordance was seen between PSQ and Next Gen-RDST when looking at additional SNPs not conferring resistance (i.e., *gyrA* 95ACC).

b *katG* resistance-conferring mutation present.

c *eis* −12T mutation present.

## DISCUSSION

Targeted NGS using amplicon sequencing direct from clinical TB samples is an open-ended (i.e., able to expand or retract targets) approach to rapid molecular DST, using a tabletop sequencer paired with an automated bioinformatic solution adapted for use on a laptop computer or cloud service. The Next Gen-RDST assay is an attractive option for the identification and characterization of drug resistance in clinical samples due to: (i) the fast turnaround time (ii) open-ended comprehensive target analyses (iii) batched (pooled) sample capability (lowering per sample cost) and (iv) the ability to provide detailed and clinically actionable information. While whole-genome sequencing allows for an examination of all mutations in the genome and provides full characterization of clinical isolates, most whole-genome sequencing approaches require close to a week of M. tuberculosis culture prior, necessitating diagnostic delays and limiting the technique to high-resource settings with access to biosafety level 3 (BSL3) culture facilities (13–16, 36, 37). The current national surveillance of TB drug resistance remains largely limited to first-line drugs; efforts by WHO have expanded surveillance for M. tuberculosis resistance to second-line drugs in only a few countries (1). All efforts to expand DST for case management and surveillance are hindered by cost and by the need for complex laboratory infrastructure, especially to support M. tuberculosis culture. TB drug resistance testing for case management and surveillance could be significantly expanded through the utilization of a targeted amplicon sequencing approach, including targets for second-line drugs; this would result in efficient and reliable drug resistance detection directly from sputum, without the need for culturing facilities.

Overall, there was high concordance between the phenotypic DST and the Next Gen-RDST analysis results (Table 4), with 25 discordances observed in 18 samples. Four samples with susceptible phenotypic DST results (21-002 with OFX; 21-0004, 22-0097, and 22-0123 with KAN) contained mutations associated with resistance that were identified by both the Next Gen-RDST and pyrosequencing assays. However, in one case (21-0004, KAN), the Next Gen-RDST assay identified a 60% mixture of resistant alleles. The presence of this subpopulation may explain an observed discordance between phenotypic and genotypic analyses. As phenotypic DST requires culture, it is conceivable that the 40% wild-type mixture was selected in culture process and grew out as susceptible in the DST process. Also, while phenotypic DST theoretically detects 1% or more of the sample population being drug resistant (38, 39), some studies have shown that subpopulations can account for discordant phenotypic DST results, with up to 10% of the resistance subpopulation going undetected (22, 25). The majority of the discordances seen here, however, were phenotypically resistant, with no resistance mutations found by genotypic methods. These discordances are unlikely the result of errors in NGS but rather reflect the true absence of a mutated genotype within the genetic regions examined, which is consistent with previous studies. The low sensitivity observed for the detection of AMK and CAP resistance was perhaps due to the low number of phenotypically resistant samples available for analysis. Other studies of tests relying upon the *rrs* 1401G mutation for AMK and CAP resistance detection have reported sensitivities as low as 57% (40); however, our analysis also included *rrs* 1484T and the *eis* promoter region, and thus, we expected higher sensitivities. In future studies, increasing the number of AMK- and CAP-resistant samples will be necessary to fully examine the strength of this assay for these drugs. There is a possibility that these observed discordances result from the failure of our assay to include additional gene regions associated with injectable resistance, such as the *tlyA* mutations (41). This emphasizes the need for further investigation into the genetic basis of drug resistance in M. tuberculosis.

Interestingly, sample 21-0073, which showed discordance with a wild-type genotype on NGS and pyrosequencing but phenotypic INH resistance, had a low-level (2%) subpopulation when we examined the detailed NGS reads for this isolate. Pyrosequencing was unable to detect evidence of mutation. For this study, a sample was called resistant by the Next Gen-RDST assay if the resistant allele was found at a frequency of ≥10%. The ability to identify mixtures of both susceptible and resistant populations and determine the threshold of what level of mixture a genotypic allele is called resistant is crucial in future molecular assays.

In a comparison of the two genotypic methods to establish drug resistance profiles, we found that indeterminate results occurred more frequently with pyrosequencing than with the Next Gen-RDST sequencing approach. When both genotyping methods had sequence data and thus allele calls, there was a high level of concordance (see Data Set S1 in the supplemental material). The genotypic method discordance (Table 5) mostly occurred in the *eis* promoter target, and in most cases, the Next Gen-RDST result was concordant with the phenotypic results and found a mutation at the −12 position, where the pyrosequencing found no mutation in the gene target (Data Set S1). One sample (22-0116) had a subpopulation with 14% of the amplicons identified as resistant by the Next Gen-RDST assay, whereas there were no mutations identified by pyrosequencing, and the sample was phenotypically resistant; this again demonstrates the importance of mixture detection and the need for molecular tools for identifying drug resistance in subpopulations. A total of four resistant alleles were identified by the Next Gen-RDST assay as minor components of the population (10 to 22%) in phenotypically resistant specimens that were not detected by pyrosequencing (Table 5). Importantly, two samples (21-0021 and 21-0004) had resistant subpopulations (10% and 16%, respectively) in the *rrs* target with the Next Gen-RDST assay, but no mutations were identified by pyrosequencing, and both were phenotypically susceptible to the injectables. Determining thresholds for establishing a “resistant” call in the presence of such mixtures is extremely important, as our observed discordance may indicate a “preresistance” state, allowing for the future development of clinically relevant phenotypic drug resistance (42–44). It is likely that these thresholds will be drug and possibly mutation dependent.

The improved sensitivity and specificity of the Next Gen-RDST assay over a well-documented sequencing approach (pyrosequencing) (31, 45–48) establishes NGS amplicon-based sequencing as a logical evolution of molecular DST. The ability of the Next Gen-RDST assay to provide improved resistance and subpopulation mixture information over both MGIT DST and pyrosequencing further establishes this method for clinically relevant characterization of M. tuberculosis infections. There are, however, multiple factors to consider when implementing such an approach, including (i) the variable nature of the bacterial load in clinical samples, (ii) the relationship of subpopulation resistance levels to eventual phenotype and clinical outcome, (iii) the fact that strain diversity in a sputum sample does not necessarily represent infection diversity in a patient, (iv) the fact that the targeted nature of the approach is complicated by the continued and evolving understanding of mutational causes of resistance, and (v) the continued reduction in length of time for sample preparation and sequence analysis.

**FIG 2:**
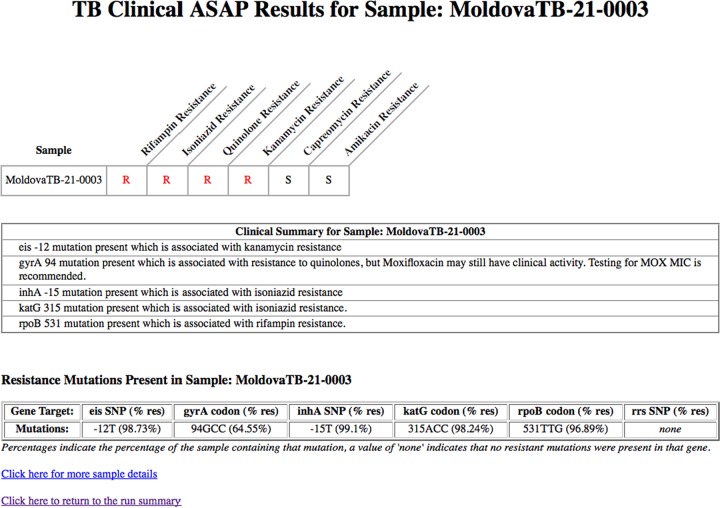
TB-ASAP clinical level output. R, resistant; S, susceptible; % res, percent resistant.

As new genes and mutations are identified and characterized, they can easily be added into the Next Gen-RDST analysis. New amplicons can be quickly designed and included in an expanded multiplex with limited cost and complications. With the TB-ASAP clinical report (Fig. 2), if new SNPs are identified and already contained in the current amplicons, simply adding the position information into the bioinformatics script allows for their inclusion into the analysis, resulting in enhanced resistance identification. This modification also allows for the analysis of previous amplicon sequence data, including the newly identified positions, with no additional laboratory work. Having the entire amplicon sequence results, instead of only select mutations of interest, allows researchers to continue characterizing other positions within the target regions. This analysis can easily be done by simply running the amplicons through an SNP discovery pipeline; this is included in the TB-ASAP tool described here. Interestingly, only six additional mutations were found when SNP identification analysis was conducted across the entire amplicon for all the targets (Table 3). Currently, databases of resistance-conferring SNPs are being curated for M. tuberculosis, which will expand the effectiveness of molecular diagnostics to include a more exhaustive set of known resistance-related positions (www.platform.reseqtb.org). For the current comparison of phenotypic DST and our Next Gen-RDST assay, we used only well-characterized SNPs that were previously identified for XDR determination (35). We found high sensitivity and specificity using this subset of resistance-conferring mutations, but as more sequence data are collected and examined, the addition of new resistance-conferring loci will help increase the sensitivity even further.

Utilizing amplicon NGS to obtain complete drug resistance profiles directly from patient samples has the potential to allow for personalized appropriate rapid treatment plans to be designed early on rather than weeks to months into an infection, potentially averting the acquisition of additional resistance. For the current assay, a clinical sample can be processed and sequenced for ∼$30 in ≤72 h (20), depending on the number of samples analyzed simultaneously. Cost and processing/sequencing time can likely be improved with additional design and process optimizations. The amplicons generated allow for the inclusion of more mutations of interest in a single amplicon, as well as the examination of novel or rare mutations within the entire amplicon. This method is also easy to expand to new targets and is already being expanded to include resistance markers to other relevant drugs (e.g., pyrazinamide). The use of TB-ASAP results in a clinician report that provides a clear summary of actionable information. In addition, the use of SMOR analysis allows for the detection and characterization of extremely low-level mixtures within the sample and will lead to a more complete understanding of disease dynamics during an infection (i.e., at what level mixtures become clinically relevant). Overall, rapid amplicon sequencing approaches, such as the Next Gen-RDST assay, should allow for appropriate treatment and easy monitoring of patients to help ensure treatment success.

## Supplementary Material

Supplemental material
