# Supplementary material for: Rapid Drug Susceptibility Testing of Drug-Resistant Mycobacterium tuberculosis Isolates Directly from Clinical Samples by Use of Amplicon Sequencing: a Proof-of-Concept Study
Source: J Clin Microbiol. 2016 Jul 25;54(8):2058–67. doi: 10.1128/JCM.00535-16 (PMC4963505; doi:10.1128/JCM.00535-16)
Supplement: Supplemental material [file supp_54_8_2058__index.html]

Supplemental material 

# Rapid Drug Susceptibility Testing of Drug-Resistant Mycobacterium tuberculosis Isolates Directly from Clinical Samples by Use of Amplicon Sequencing: a Proof-of-Concept Study

## Supplemental material

- Supplemental file 1 -

  Table S1 (Universal tail 2 primer sequences)

  PDF, 56K
- Supplemental file 2 -

  Data Set S1 (Resistance versus susceptibility calls across three platforms)

  XLSX, 86K
