# Supplementary material for: Rapid Drug Susceptibility Testing of Drug-Resistant Mycobacterium tuberculosis Isolates Directly from Clinical Samples by Use of Amplicon Sequencing: a Proof-of-Concept Study
Source: J Clin Microbiol. 2016 Jul 25;54(8):2058–67. doi: 10.1128/JCM.00535-16 (PMC4963505; doi:10.1128/JCM.00535-16)
Supplement: Supplemental material [file JCM.00535-16_zjm999095082so1.pdf]

TABLE S1: Universal Tail (UT2) primer sequences

Primer name	Primer sequence
Indexed-UT2-8bp1	CAAGCAGAAGACGGCATACGAGATACAAGCTAAGTCAGTCAGCCACGCACTTGACTTGTCTTC
Indexed-UT2-8bp10	CAAGCAGAAGACGGCATACGAGATAGTACAAGAGTCAGTCAGCCACGCACTTGACTTGTCTTC
Indexed-UT2-8bp11	CAAGCAGAAGACGGCATACGAGATCATCAAGTAGTCAGTCAGCCACGCACTTGACTTGTCTTC
Indexed-UT2-8bp12	CAAGCAGAAGACGGCATACGAGATAGTGGTCAAGTCAGTCAGCCACGCACTTGACTTGTCTTC
Indexed-UT2-8bp13	CAAGCAGAAGACGGCATACGAGATAACAACCAAGTCAGTCAGCCACGCACTTGACTTGTCTTC
Indexed-UT2-8bp14	CAAGCAGAAGACGGCATACGAGATAACCGAGAAGTCAGTCAGCCACGCACTTGACTTGTCTTC
Indexed-UT2-8bp15	CAAGCAGAAGACGGCATACGAGATAACGCTTAAGTCAGTCAGCCACGCACTTGACTTGTCTTC
Indexed-UT2-8bp16	CAAGCAGAAGACGGCATACGAGATAAGACGGAAGTCAGTCAGCCACGCACTTGACTTGTCTTC
Indexed-UT2-8bp17	CAAGCAGAAGACGGCATACGAGATAAGGTACAAGTCAGTCAGCCACGCACTTGACTTGTCTTC
Indexed-UT2-8bp18	CAAGCAGAAGACGGCATACGAGATACACAGAAAGTCAGTCAGCCACGCACTTGACTTGTCTTC
Indexed-UT2-8bp19	CAAGCAGAAGACGGCATACGAGATACAGCAGAAGTCAGTCAGCCACGCACTTGACTTGTCTTC
Indexed-UT2-8bp2	CAAGCAGAAGACGGCATACGAGATAAACATCGAGTCAGTCAGCCACGCACTTGACTTGTCTTC
Indexed-UT2-8bp20	CAAGCAGAAGACGGCATACGAGATACCTCCAAAGTCAGTCAGCCACGCACTTGACTTGTCTTC
Indexed-UT2-8bp21	CAAGCAGAAGACGGCATACGAGATACGCTCGAAGTCAGTCAGCCACGCACTTGACTTGTCTTC
Indexed-UT2-8bp22	CAAGCAGAAGACGGCATACGAGATACGTATCAAGTCAGTCAGCCACGCACTTGACTTGTCTTC
Indexed-UT2-8bp23	CAAGCAGAAGACGGCATACGAGATACTATGCAAGTCAGTCAGCCACGCACTTGACTTGTCTTC
Indexed-UT2-8bp24	CAAGCAGAAGACGGCATACGAGATAGAGTCAAAGTCAGTCAGCCACGCACTTGACTTGTCTTC
Indexed-UT2-8bp25	CAAGCAGAAGACGGCATACGAGATAGATCGCAAGTCAGTCAGCCACGCACTTGACTTGTCTTC
Indexed-UT2-8bp26	CAAGCAGAAGACGGCATACGAGATAGCAGGAAAGTCAGTCAGCCACGCACTTGACTTGTCTTC
Indexed-UT2-	CAAGCAGAAGACGGCATACGAGATAGTCACTAAGTCAGTCAGCCACGCACTTGACT

8bp27	TGTCTTC
Indexed-UT2-8bp28	CAAGCAGAAGACGGCATACGAGATATCCTGTAAGTCAGTCAGCCACGCACTTGACT TGTCTTC
Indexed-UT2-8bp29	CAAGCAGAAGACGGCATACGAGATATTGAGGAAGTCAGTCAGCCACGCACTTGACT TGTCTTC
Indexed-UT2-8bp3	CAAGCAGAAGACGGCATACGAGATACATTGGCAGTCAGTCAGCCACGCACTTGACT TGTCTTC
Indexed-UT2-8bp30	CAAGCAGAAGACGGCATACGAGATCAACCACAAGTCAGTCAGCCACGCACTTGACT TGTCTTC
Indexed-UT2-8bp31	CAAGCAGAAGACGGCATACGAGATCAAGACTAAGTCAGTCAGCCACGCACTTGACT TGTCTTC
Indexed-UT2-8bp32	CAAGCAGAAGACGGCATACGAGATCAATGGAAAGTCAGTCAGCCACGCACTTGACT TGTCTTC
Indexed-UT2-8bp33	CAAGCAGAAGACGGCATACGAGATCACTTCGAAGTCAGTCAGCCACGCACTTGACT TGTCTTC
Indexed-UT2-8bp34	CAAGCAGAAGACGGCATACGAGATCAGCGTTAAGTCAGTCAGCCACGCACTTGACT TGTCTTC
Indexed-UT2-8bp35	CAAGCAGAAGACGGCATACGAGATCATACCAAAGTCAGTCAGCCACGCACTTGACT TGTCTTC
Indexed-UT2-8bp36	CAAGCAGAAGACGGCATACGAGATCCAGTTCAAGTCAGTCAGCCACGCACTTGACT TGTCTTC
Indexed-UT2-8bp37	CAAGCAGAAGACGGCATACGAGATCCGAAGTAAGTCAGTCAGCCACGCACTTGACT TGTCTTC
Indexed-UT2-8bp38	CAAGCAGAAGACGGCATACGAGATCCGTGAGAAGTCAGTCAGCCACGCACTTGACT TGTCTTC
Indexed-UT2-8bp39	CAAGCAGAAGACGGCATACGAGATCCTCCTGAAGTCAGTCAGCCACGCACTTGACT TGTCTTC
Indexed-UT2-8bp4	CAAGCAGAAGACGGCATACGAGATACCACTGTAGTCAGTCAGCCACGCACTTGACT TGTCTTC
Indexed-UT2-8bp40	CAAGCAGAAGACGGCATACGAGATCGAACTTAAGTCAGTCAGCCACGCACTTGACT TGTCTTC
Indexed-UT2-8bp41	CAAGCAGAAGACGGCATACGAGATCGACTGGAAGTCAGTCAGCCACGCACTTGACT TGTCTTC
Indexed-UT2-8bp42	CAAGCAGAAGACGGCATACGAGATCGCATACAAGTCAGTCAGCCACGCACTTGACT TGTCTTC
Indexed-UT2-8bp43	CAAGCAGAAGACGGCATACGAGATCTCAATGAAGTCAGTCAGCCACGCACTTGACT TGTCTTC
Indexed-UT2-8bp44	CAAGCAGAAGACGGCATACGAGATCTGAGCCAAGTCAGTCAGCCACGCACTTGACT TGTCTTC
Indexed-UT2-8bp45	CAAGCAGAAGACGGCATACGAGATCTGGCATAAGTCAGTCAGCCACGCACTTGACT TGTCTTC
Indexed-UT2-8bp46	CAAGCAGAAGACGGCATACGAGATGAATCTGAAGTCAGTCAGCCACGCACTTGACT TGTCTTC

Indexed-UT2-8bp47	CAAGCAGAAGACGGCATACGAGATGACTAGTAAGTCAGTCAGCCACGCACTTGACT TGTCTTC
Indexed-UT2-8bp48	CAAGCAGAAGACGGCATACGAGATGAGCTGAAAGTCAGTCAGCCACGCACTTGACT TGTCTTC
Indexed-UT2-8bp49	CAAGCAGAAGACGGCATACGAGATGATAGACAAGTCAGTCAGCCACGCACTTGACT TGTCTTC
Indexed-UT2-8bp5	CAAGCAGAAGACGGCATACGAGATAACGTGATAGTCAGTCAGCCACGCACTTGACT TGTCTTC
Indexed-UT2-8bp50	CAAGCAGAAGACGGCATACGAGATGCCACATAAGTCAGTCAGCCACGCACTTGACT TGTCTTC
Indexed-UT2-8bp51	CAAGCAGAAGACGGCATACGAGATGCGAGTAAAGTCAGTCAGCCACGCACTTGACT TGTCTTC
Indexed-UT2-8bp52	CAAGCAGAAGACGGCATACGAGATGCTAACGAAGTCAGTCAGCCACGCACTTGACT TGTCTTC
Indexed-UT2-8bp53	CAAGCAGAAGACGGCATACGAGATGCTCGGTAAGTCAGTCAGCCACGCACTTGACT TGTCTTC
Indexed-UT2-8bp54	CAAGCAGAAGACGGCATACGAGATGGAGAACAAGTCAGTCAGCCACGCACTTGAC TTGTCTTC
Indexed-UT2-8bp55	CAAGCAGAAGACGGCATACGAGATGGTGCGAAAGTCAGTCAGCCACGCACTTGACT TGTCTTC
Indexed-UT2-8bp56	CAAGCAGAAGACGGCATACGAGATGTACGCAAAGTCAGTCAGCCACGCACTTGACT TGTCTTC
Indexed-UT2-8bp57	CAAGCAGAAGACGGCATACGAGATGTCGTAGAAGTCAGTCAGCCACGCACTTGACT TGTCTTC
Indexed-UT2-8bp58	CAAGCAGAAGACGGCATACGAGATGTCTGTCAAGTCAGTCAGCCACGCACTTGACT TGTCTTC
Indexed-UT2-8bp59	CAAGCAGAAGACGGCATACGAGATGTGTTCTAAGTCAGTCAGCCACGCACTTGACT TGTCTTC
Indexed-UT2-8bp6	CAAGCAGAAGACGGCATACGAGATCGCTGATCAGTCAGTCAGCCACGCACTTGACT TGTCTTC
Indexed-UT2-8bp60	CAAGCAGAAGACGGCATACGAGATTAGGATGAAGTCAGTCAGCCACGCACTTGACT TGTCTTC
Indexed-UT2-8bp61	CAAGCAGAAGACGGCATACGAGATTATCAGCAAGTCAGTCAGCCACGCACTTGACT TGTCTTC
Indexed-UT2-8bp62	CAAGCAGAAGACGGCATACGAGATTCCGTCTAAGTCAGTCAGCCACGCACTTGACT TGTCTTC
Indexed-UT2-8bp63	CAAGCAGAAGACGGCATACGAGATTCTTCACAAGTCAGTCAGCCACGCACTTGACTT GTCTTC
Indexed-UT2-8bp64	CAAGCAGAAGACGGCATACGAGATTGAAGAGAAGTCAGTCAGCCACGCACTTGACT TGTCTTC
Indexed-UT2-8bp65	CAAGCAGAAGACGGCATACGAGATTGGAACAAAGTCAGTCAGCCACGCACTTGACT TGTCTTC
Indexed-UT2-	CAAGCAGAAGACGGCATACGAGATTGGCTTCAAGTCAGTCAGCCACGCACTTGACT

8bp66	TGTCTTC
Indexed-UT2-8bp67	CAAGCAGAAGACGGCATACGAGATTGGTGGTAAGTCAGTCAGCCACGCACTTGACTTGTCTTC
Indexed-UT2-8bp68	CAAGCAGAAGACGGCATACGAGATTTCACGCAAGTCAGTCAGCCACGCACTTGACTTGTCTTC
Indexed-UT2-8bp69	CAAGCAGAAGACGGCATACGAGATAACTCACCAGTCAGTCAGCCACGCACTTGACTTGTCTTC
Indexed-UT2-8bp7	CAAGCAGAAGACGGCATACGAGATCAGATCTGAGTCAGTCAGCCACGCACTTGACTTGTCTTC
Indexed-UT2-8bp70	CAAGCAGAAGACGGCATACGAGATAAGAGATCAGTCAGTCAGCCACGCACTTGACTTGTCTTC
Indexed-UT2-8bp71	CAAGCAGAAGACGGCATACGAGATAAGGACACAGTCAGTCAGCCACGCACTTGACTTGTCTTC
Indexed-UT2-8bp72	CAAGCAGAAGACGGCATACGAGATAATCCGTCAGTCAGTCAGCCACGCACTTGACTTGTCTTC
Indexed-UT2-8bp73	CAAGCAGAAGACGGCATACGAGATAATGTTGCAGTCAGTCAGCCACGCACTTGACTTGTCTTC
Indexed-UT2-8bp74	CAAGCAGAAGACGGCATACGAGATACACGACCAGTCAGTCAGCCACGCACTTGACTTGTCTTC
Indexed-UT2-8bp75	CAAGCAGAAGACGGCATACGAGATACAGATTCAGTCAGTCAGCCACGCACTTGACTTGTCTTC
Indexed-UT2-8bp76	CAAGCAGAAGACGGCATACGAGATAGATGTACAGTCAGTCAGCCACGCACTTGACTTGTCTTC
Indexed-UT2-8bp77	CAAGCAGAAGACGGCATACGAGATAGCACCTCAGTCAGTCAGCCACGCACTTGACTTGTCTTC
Indexed-UT2-8bp78	CAAGCAGAAGACGGCATACGAGATAGCCATGCAGTCAGTCAGCCACGCACTTGACTTGTCTTC
Indexed-UT2-8bp79	CAAGCAGAAGACGGCATACGAGATAGGCTAACAGTCAGTCAGCCACGCACTTGACTTGTCTTC
Indexed-UT2-8bp8	CAAGCAGAAGACGGCATACGAGATATGCCTAAAGTCAGTCAGCCACGCACTTGACTTGTCTTC
Indexed-UT2-8bp80	CAAGCAGAAGACGGCATACGAGATATAGCGACAGTCAGTCAGCCACGCACTTGACTTGTCTTC
Indexed-UT2-8bp81	CAAGCAGAAGACGGCATACGAGATATCATTCCAGTCAGTCAGCCACGCACTTGACTTGTCTTC
Indexed-UT2-8bp82	CAAGCAGAAGACGGCATACGAGATATTGGCTCAGTCAGTCAGCCACGCACTTGACTTGTCTTC
Indexed-UT2-8bp83	CAAGCAGAAGACGGCATACGAGATCAAGGAGCAGTCAGTCAGCCACGCACTTGACTTGTCTTC
Indexed-UT2-8bp84	CAAGCAGAAGACGGCATACGAGATCACCTTACAGTCAGTCAGCCACGCACTTGACTTGTCTTC
Indexed-UT2-8bp85	CAAGCAGAAGACGGCATACGAGATCCATCCTCAGTCAGTCAGCCACGCACTTGACTTGTCTTC

Indexed-UT2-8bp86	CAAGCAGAAGACGGCATACGAGATCCGACAACAGTCAGTCAGCCACGCACTTGACTTGTCTTC
Indexed-UT2-8bp87	CAAGCAGAAGACGGCATACGAGATCCTAATCCAGTCAGTCAGCCACGCACTTGACTTGTCTTC
Indexed-UT2-8bp88	CAAGCAGAAGACGGCATACGAGATCCTCTATCAGTCAGTCAGCCACGCACTTGACTTGTCTTC
Indexed-UT2-8bp89	CAAGCAGAAGACGGCATACGAGATCGACACACAGTCAGTCAGCCACGCACTTGACTTGTCTTC
Indexed-UT2-8bp90	CAAGCAGAAGACGGCATACGAGATCGGATTGCAGTCAGTCAGCCACGCACTTGACTTGTCTTC
Indexed-UT2-8bp91	CAAGCAGAAGACGGCATACGAGATCTAAGGTCAGTCAGTCAGCCACGCACTTGACTTGTCTTC
Indexed-UT2-8bp92	CAAGCAGAAGACGGCATACGAGATGAACAGGCAGTCAGTCAGCCACGCACTTGACTTGTCTTC
Indexed-UT2-8bp93	CAAGCAGAAGACGGCATACGAGATGACAGTGCAGTCAGTCAGCCACGCACTTGACTTGTCTTC
Indexed-UT2-8bp94	CAAGCAGAAGACGGCATACGAGATGAGTTAGCAGTCAGTCAGCCACGCACTTGACTTGTCTTC
Indexed-UT2-8bp95	CAAGCAGAAGACGGCATACGAGATGATGAATCAGTCAGTCAGCCACGCACTTGACTTGTCTTC
Indexed-UT2-8bp96	CAAGCAGAAGACGGCATACGAGATGCCAAGACAGTCAGTCAGCCACGCACTTGACTTGTCTTC
